# Developing a classification system to assign activity states to two species of freshwater turtles

**DOI:** 10.1371/journal.pone.0277491

**Published:** 2022-11-30

**Authors:** Anne-Christine Auge, Gabriel Blouin-Demers, Dennis L. Murray

**Affiliations:** 1 Department of Biology, Trent University, Peterborough, ON, Canada; 2 Department of Biology, University of Ottawa, Ottawa, ON, Canada; Wildlife Conservation Society Canada, CANADA

## Abstract

Research in ecology often requires robust assessment of animal behaviour, but classifying behavioural patterns in free-ranging animals and in natural environments can be especially challenging. New miniaturised bio-logging devices such as accelerometers are increasingly available to record animal behaviour remotely, and thereby address the gap in knowledge related to behaviour of free-ranging animals. However, validation of these data is rarely conducted and classification model transferability across closely-related species is often not tested. Here, we validated accelerometer and water sensor data to classify activity states in two free-ranging freshwater turtle species (Blanding’s turtle, *Emydoidea blandingii*, and Painted turtle, *Chrysemys picta*). First, using only accelerometer data, we developed a decision tree to separate motion from motionless states, and second, we included water sensor data to classify the animal as being motionless or in-motion on land or in water. We found that accelerometers separated in-motion from motionless behaviour with > 83% accuracy, whereas models also including water sensor data predicted states in terrestrial and aquatic locations with > 77% accuracy. Despite differences in values separating activity states between the two species, we found high model transferability allowing cross-species application of classification models. Note that reducing sampling frequency did not affect predictive accuracy of our models up to a sampling frequency of 0.0625 Hz. We conclude that the use of accelerometers in animal research is promising, but requires prior data validation and development of robust classification models, and whenever possible cross-species assessment should be conducted to establish model generalisability.

## Introduction

Advances in behavioural ecology often depend on effectively quantifying activity and behaviours in free-ranging animals [1, 2]. For example, closely-related species with overlapping ranges may co-exist through a variety of mechanisms including resource partitioning through differing activity patterns or space use [3, 4]. Behavioural syndromes (e.g., bold/shy classification) can help explain factors such as individual behavioural responses to anthropogenic or environmental stressors, and often require quantification of behavioural response intensity across a stress gradient (e.g. [5]). Likewise, knowledge of behavioural responses of individuals can guide management decisions for species-at-risk such as design of dispersal corridors (e.g. [6])or establishment of captive breeding programs (e.g. [7]). Accordingly, collecting robust, fine-scale activity and behavioural data should be a high priority in ecology and conservation biology.

Traditionally, activity and behavioural data are collected via direct observation of captive (e.g. [8]) and wild (e.g. [9]) animals, or via a variety of remote-monitoring technologies such as radio or acoustic telemetry [10, 11]. These traditional methods, however, can be imprecise and possibly biased due to coarse or inaccurate data [12, 13]. For elusive species, traditional measurements may also yield fragmented data and thus be of limited use for quantifying sources of variation in behaviour. However, new miniaturized bio-logging tools may be particularly useful for monitoring activity and behaviour of cryptic species or those living in inaccessible habitats if they can characterize activity and behaviours at a scale and level of precision that is commensurate with contemporary research questions. In particular, modern bio-loggers record information about animal location, body position, or physiology continuously and at a very fine scale (e.g. [14–16]). Global positioning system (GPS) devices and accelerometers are now commonly deployed on wild animals and are often coupled with different environmental sensors such as thermometers or magnetometers [17, 18]. In the last two decades, accelerometry has become increasingly popular for studying animal activity, behaviour, and energy expenditure [19] by recording high-resolution body acceleration in up to three dimensions (see S1 Fig) and thereby providing information about animal posture and proxies for activity levels [20, 21]. Accelerometer-derived movement and posture data, in turn, can inform about animal behaviour and activity states [22, 23]. Thus, acceleration data, alone or in combination with data from other sensors, can assign or classify behaviours, including across a variety of animals and settings [24, 25]. Several methods have been used to translate acceleration data into behaviour, including unsupervised machine learning approaches which use complex algorithms to find patterns in unlabeled datasets from which behaviour can be subjectively inferred [24, 26], and computer models that are objectively trained to classify behaviour using labelled data and acceleration thresholds (e.g. classification models or decision trees) [27–29].

Despite the frequent use of accelerometry, many studies use subjective assessment of accelerometry data to infer behaviour without proper validation. It is understood that validating accelerometer measurements can be onerous given that data need to be matched to known behaviours [26, 29], and obtaining representative behavioural data from free-ranging animals can be especially challenging. Captive animals in unnatural settings often are more readily available for behavioural trials, but the extent that data collected under these conditions are relatable to behaviours of free-ranging animals is questionable [30, 31]. Nevertheless, studies using accelerometers are compelled to develop plans for data validation and classification during early phases of a study [31].

Accelerometer signatures can vary according to body size, shape and movement patterns, meaning that species may differ in their acceleration signature when performing similar behaviours. Accordingly, use of behavioural classification models developed for one species on another may not be appropriate, especially without prior validation [22]. However, in theory, closely-related species may yield similar accelerometer readings when performing similar behaviours, and therefore cross-species validation may be relevant in some cases. It follows that using a single classification model for similar species could improve and streamline classification and accelerate broad adoption of accelerometers in behaviour research. Thus, understanding behaviour classification model transferability across similar species should be prioritized ([29], but see [32]). Another approach to improve efficiencies in accelerometer studies is to reduce sampling frequency [27, 33]. As a framework for collecting reliable activity and behavioural data from accelerometers, it is important to use device programming schedules in accordance with the body size and ecology of target species [30, 34], with slower moving animals or those with simpler behavioural repertoires potentially receiving accelerometers programmed with a lower sampling frequency [24, 35, 36].

**Fig 1:**
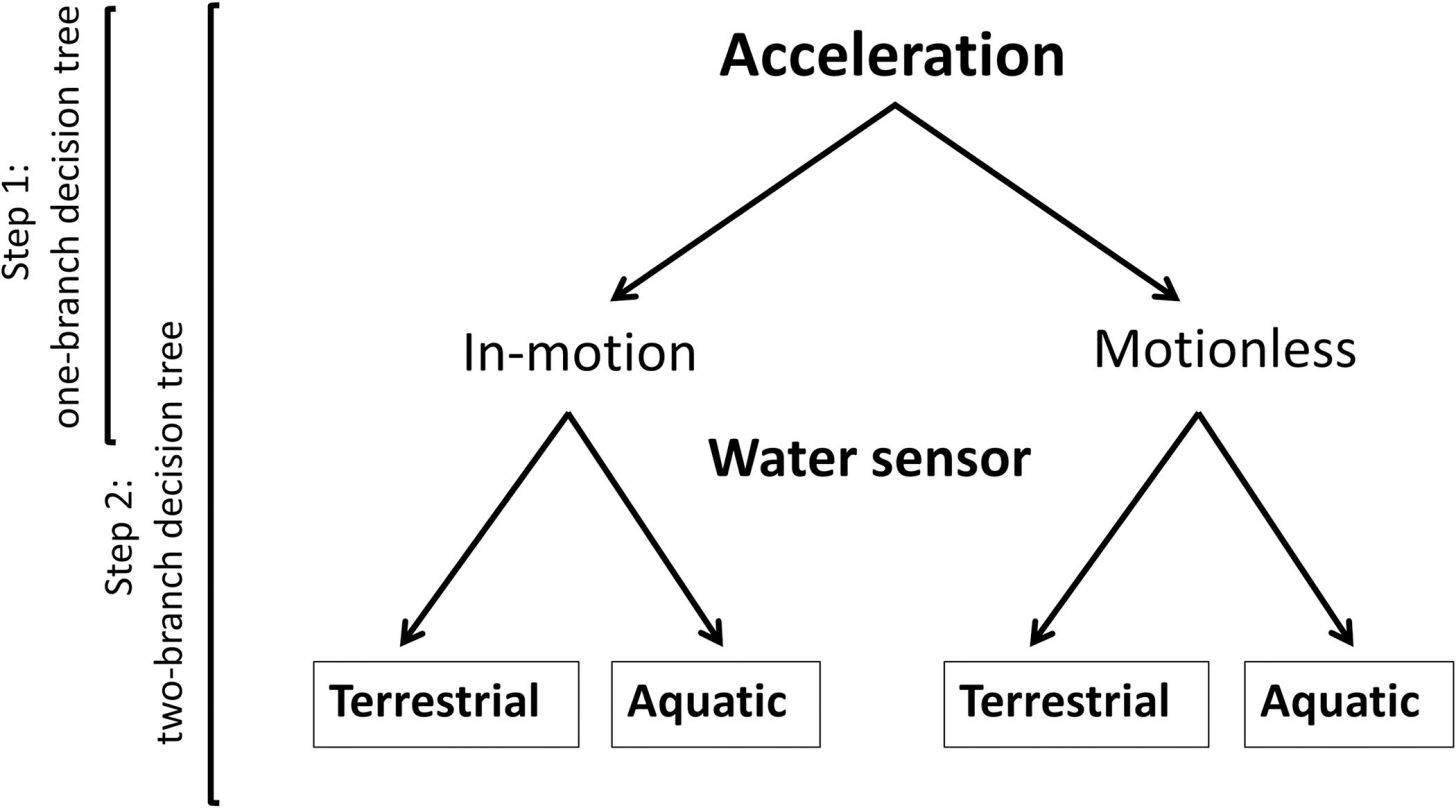
Two-step decision tree for classifying the main activities of freshwater turtles. Acceleration data are first binned according to activity level (Step 1) based on visual observation and acceleration thresholds, and then further classified according to habitat type based on a water conductivity sensor deployed in tandem with the accelerometer (Step 2). The same process was used to classify activity in both turtle species, resulting in four categories.

In this study, we used accelerometers and water sensors to develop a behaviour classification model for two free-ranging sympatric freshwater turtles: Blanding’s turtles (*Emydoidea blandingii*) and Painted turtles (*Chrysemys picta*). These turtles have similar life history and habitat requirements, and co-occur in shallow ponds and marshes across eastern North America [37, 38]. Both species spend considerable time basking or under water, and use terrestrial habitat to varying degrees when travelling between wetlands and for nesting [39]. First, we demonstrate the process of developing and validating a robust classification model for turtle activity states, visualised as a decision tree (Fig 1), and compared the performance of classification models for each species, based on acceleration signatures. Second, we assessed the transferability of our species-specific classification models via cross-species comparison. We also explored the optimisation of accelerometer programming by assessing the role of sampling frequency on classification accuracy. Finally, we illustrate the application of our behaviour classification by reporting daily activity-budgets of free-ranging Blanding’s and Painted turtles.

## Materials and methods

We manually developed and validated a classification model for Blanding’s and Painted turtle activity states using the following steps: a) matching observed activity states in the wild to recorded acceleration data, b) evaluation of smoothing window to calculate various acceleration metrics, c) determination of the acceleration metric that best separated activity states using histogram separation, d) determination of thresholds separating activity states using histogram separation and performance measures, and e) calculation of overall model performance using both accelerometer and water sensor data. We selected a manual approach to behavioural classification to clearly demonstrate each step necessary to assign behaviours based on accelerometer data. This approach is often perceived as more comprehensible, making it more easily transferable to a variety of research settings [27, 29].

### Field methods and data collection

We studied Blanding’s and Painted turtles in the South March Highlands Conservation Forest in Ottawa, Ontario, Canada (45°20ˈ N, 75°56’ W) in the summers 2018–2020. Turtles (Blanding’s (n = 16); Painted (n = 23)) were captured using baited hoop-nets or by hand and fitted with a GPS/tri-axial accelerometer data logger (model AxyTrek, Technosmart, Rome, Italy) and VHF transmitter (model SI-2, Holohil, Carp, Canada) bolted to the carapace margin (9th to 11th scute), respectively (S2 Fig). Both units comprised < 10% of turtle body mass and position of loggers was kept constant to ensure comparability. Data loggers recorded water conductivity and acceleration at a frequency of 1 Hz (10 bit resolution, ± 2 g_force_). For activity classification and validation, videos of 8 wild Blanding’s and 9 wild Painted turtles were recorded (range: 1 min 57 s to 23 min 38 s) with a Smartphone camera (Motorola Moto G6). Turtles were recorded after being released at the capture site following a 20–30 min recovery period, and were tracked until they were out of sight. This mostly occurred when animals disappeared in deeper or densely vegetated water. During recordings, we remained distant from the animals to avoid disturbing their natural behaviour and rarely censored observations that were notably influenced by our activities. We deemed observed behaviours as being natural because they were comparable to those we observed in other wild turtles that we did not handle. All turtles were re-captured at the end of each summer to retrieve data loggers. All animals were handled in accordance with guidelines from the Canadian Council on Animal Care (CCAC) (2005) and procedures were approved by the Trent University Animal Care Committee (Protocol No. 24729) and by the Ministry of Natural Resources and Forestry (MNRF, Permit No. KV-C-002-14).

### Activity annotation and time synchronisation

Using video footage recorded in the field, we categorized turtle activity per second. We observed the following behaviours: locomotion (walking and swimming, hereafter referred to as “terrestrial in-motion” or “aquatic in-motion”, respectively), defined as forward movement lasting longer than 2 s, and motionless activity (turtles immobile out of water, hereafter referred to as “terrestrial-motionless”, and sitting or floating in water, hereafter referred to as “aquatic-motionless”). Annotating acceleration data with activity using video and external time devices introduces potential time synchronisation errors [27]. We synchronised start and end time of videos with accelerometer time (received from satellite systems) and time noted on an Android GPS app (GPS test, Chartcross Limited). Additionally, we compared time-specific repetitive motion signatures on accelerometers recorded before deployment to the GPS time app. These signatures consisted of 30 s shaking and 30 s lying still on the ground and are visualised by plotting acceleration data. Finally, to confirm that activity annotation based on videos aligned with accelerometer time, we investigated abrupt transition in observed activity states (e.g., motionless to in-motion) in each individual and corrected time, if necessary [27]. To avoid time synchronisation uncertainty, we excluded the first and last second of each activity bout from analysis, and also censored bouts < 2 s.

### Smoothing window sensitivity analysis

Various metrics of total acceleration (dynamic body acceleration, DBA, see below) can be calculated from raw tri-axial acceleration data. DBA represents average raw acceleration in each body axis over time, resulting in static acceleration, which is subtracted from raw acceleration, yielding the dynamic portion caused by movement [19]. The averaging window is dependent on stroke duration and DBA sensitivity should be assessed relative to duration of the smoothing window [35]. The first step in the development of behavioural classification models, is to determine the suitable smoothing window to calculate DBA metrics. Thus, we investigated overall dynamic body acceleration (ODBA, see below) variation derived from running median durations ranging from 3 to 131 s using data from video-recorded trials for each activity mode and species separately [35]. We visually inspected ODBA plots and selected the smoothing window with lowest ODBA variability [35]. We then calculated the greatest mean ODBA value within 95% of the maximum and chose the corresponding smoothing window. A two-tailed paired t-test [35] served to determine if ODBA values differed between selected windows and the next longest window.

### Calculation of acceleration metrics

We calculated six DBA metrics known to be relevant to activity and behavioural classification (e.g. [27, 33, 40]):

1. Total overall dynamic body acceleration (ODBA), as:
$$TODBA=\sum_{i=1}^{t}\left|X_{d,i}+Y_{d,i}+Z_{d,i}\right|$$
2. Total vectorial dynamic body acceleration (VeDBA), as:
$$TVeDBA=\sum_{i=1}^{t}\sqrt{\left(X_{d,i}^{2}+Y_{d,i}^{2}+Z_{d,i}^{2}\right)}$$
3. Delta ODBA, as:
$$\Delta ODBA=\sum_{i=1}^{t}\left|{(X}_{d,i+1}-X_{d,i})+{(Y}_{d,i+1}-Y_{d,i})+{(Z}_{d,i+1}{-Z}_{d,i})\right|$$
4. Delta VeDBA, as:
$$\Delta VeDBA=\sum_{i=1}^{t}\sqrt{{{(X}_{d,i+1}-X{}_{d,i})}^{2}+{{(Y}_{d,i+1}-Y_{d,i})}^{2}+{{(Z}_{d,i+1}{-Z}_{d,i})}^{2}}$$
5. Standard deviation of ODBA, as:
$$SDODBA=\sigma {\left(|X_{d,i}+Y_{d,i}+Z_{d,i}|\right)}_{i=1}^{t}$$
6. Standard deviation of VeDBA, as:
$$SVeDBA=\sigma {\left(\sqrt{\left(X_{d,i}^{2}+Y_{d,i}^{2}+Z_{d,i}^{2}\right)}\right)}_{i=1,}^{t}$$
  where *X*_*d*,*i*_, *Y*_*d*,*i*_ and *Z*_*d*,*i*_ are dynamic accelerations in each direction at time *i*, *t* is the sampling window and *σ* is standard deviation. The sampling window of 10 s was based on the shortest mean duration of each natural activity bout, ensuring sufficient resolution [27].

### Metric and threshold value selection

To determine the DBA metric and DBA values that best separate activity states (i.e. thresholds), we used histogram separation. We randomly divided the entire dataset (combined individuals per species) with annotated behaviours into training (70%) and testing (30%), each including similar ratios of the four activity states (see [41]), using the *dplyr* package in R [42]. In the training dataset, all six DBA metrics were calculated per individual using the selected smoothing window. For each species, these DBA metrics were used to separate in-motion from motionless states in known habitats (i.e. separately for aquatic and terrestrial) (Fig 1). We plotted histograms of each DBA metric for each pair of states (terrestrial in-motion vs. motionless; aquatic in-motion vs. motionless) and calculated percent overlap between states [29]. The appropriate metric was chosen based on how clearly they separated target states. Based on histograms of the chosen metric, we calculated the following classification performance metrics for each potential threshold within the overlapping ranges, in 0.1 increments [29], using the R package *caret* [43]: sensitivity, as the proportion of instances when a certain activity state was correctly classified as having occurred out of all instances of when this activity truly occurred (TP / (TP + FN)); specificity, as the proportion of instances when an activity state did not occur and was correctly classified as not occurring (TN / (TN + FP)); and accuracy, as the instances of correct classification of activity states out of all classifications ((TP + TN) / (TP + TN + FN + FP); where TP = true positive, TN = true negative, FP = false positive, and FN = false negative [43–45]. The point where all three performance metrics were highest was chosen as the appropriate threshold value, i.e. the DBA value that best separated pairs of activity states (in-motion vs. motionless). Next, we assessed a two-step decision tree using the DBA thresholds determined in the first step and also included water sensor data to determine if activity occurred in terrestrial (water sensor ≤ 500 V) or aquatic habitat (> 500 V). The 500 V threshold was determined by separate trials involving leaving transmitters in and out of water (A. Auge, unpubl.). Finally, from a decision tree that combined the two steps (Fig 1), we calculated confusion matrices to evaluate classification performance and calculated sensitivity, specificity, and accuracy of state assignments to the test dataset based on threshold DBA values and water sensor data (Fig 1) [23]. The classification model was developed manually in R version 4.0.2 [46]; confusion matrices and measures of accuracy were calculated using the R package *caret* [43].

### Species comparison

We assessed the transferability of our classification system by testing classification performance using parameters from the Blanding’s turtle classification model on Painted turtle data, and *vice versa*: We used the smoothing window from one species to calculate acceleration metrics and find optimal threshold values for the other species, and used threshold values determined from the training dataset of one species to determine accuracy, sensitivity and specificity in classifying activity for the test dataset of the other species.

### Effect of sampling frequency

We assessed how recording frequency affects the classification model by rarefying the original acceleration dataset and selecting every 2^nd^, 4^th^, 8^th^ and 16^th^ data point to simulate a sampling frequency of 0.5, 0.25, 0.125 and 0.0625 Hz, respectively (see e.g. [33, 47, 48]). We then repeated the steps described previously for 1 Hz: selecting the appropriate smoothing window, determining the best DBA metric and thresholds via histogram separation, and calculating accuracy measurements.

### Activity-budgets

To illustrate the application and type of inference possible from accelerometer-based activity classification for free-ranging freshwater turtles, we used the thresholds determined by the classification models to calculate average daily activity-budgets for each monitored turtle during 2018–2020 as the mean proportion of a day spent expressing each activity. Because we expected differences in the behaviour between the two species due to their morphological and ecological differences [37, 38], we compared activity-budgets between species. For this comparison we used a Dirichlet regression, which accounts for the compositional characteristics of the activity-budgets [49] using the *DirichletReg* package [42] in R, where proportion of time spent in each state and species were the response and predictor variables, respectively. Note that we also performed this analysis using a non-parametric PERMANOVA, which yielded qualitatively similar results; herein we report exclusively the parametric results. All analyses were performed using R version 4.0.2 (R Development Core Team, Vienna, Austria, 2020).

## Results

### Turtle video observations

After censoring accelerometer data, we had 47 min 8 s (range: 57 s– 19 min 18 s per individual) and 73 min 3 s (range: 54 s – 16 min 52 s per individual) of activity data from Blanding’s and Painted turtles, respectively. All four pre-defined activity categories were recorded during the video trials, with Blanding’s turtle terrestrial in-motion and terrestrial-motionless being observed most frequently (55.3% and 28.5% of video minutes, respectively), followed by aquatic-motionless (9.8%) and aquatic in-motion (6.4%). In Painted turtles, terrestrial-motionless was observed most frequently (85.1%), followed by aquatic-motionless (7.1%), terrestrial in-motion (4.4%), and aquatic in-motion (3.4%) (S3 Fig). We note that, despite our best efforts, we did not observe more complex behaviours (e.g. nesting, mating, foraging) in our free-ranging study animals.

### Smoothing window

**Fig 2:**
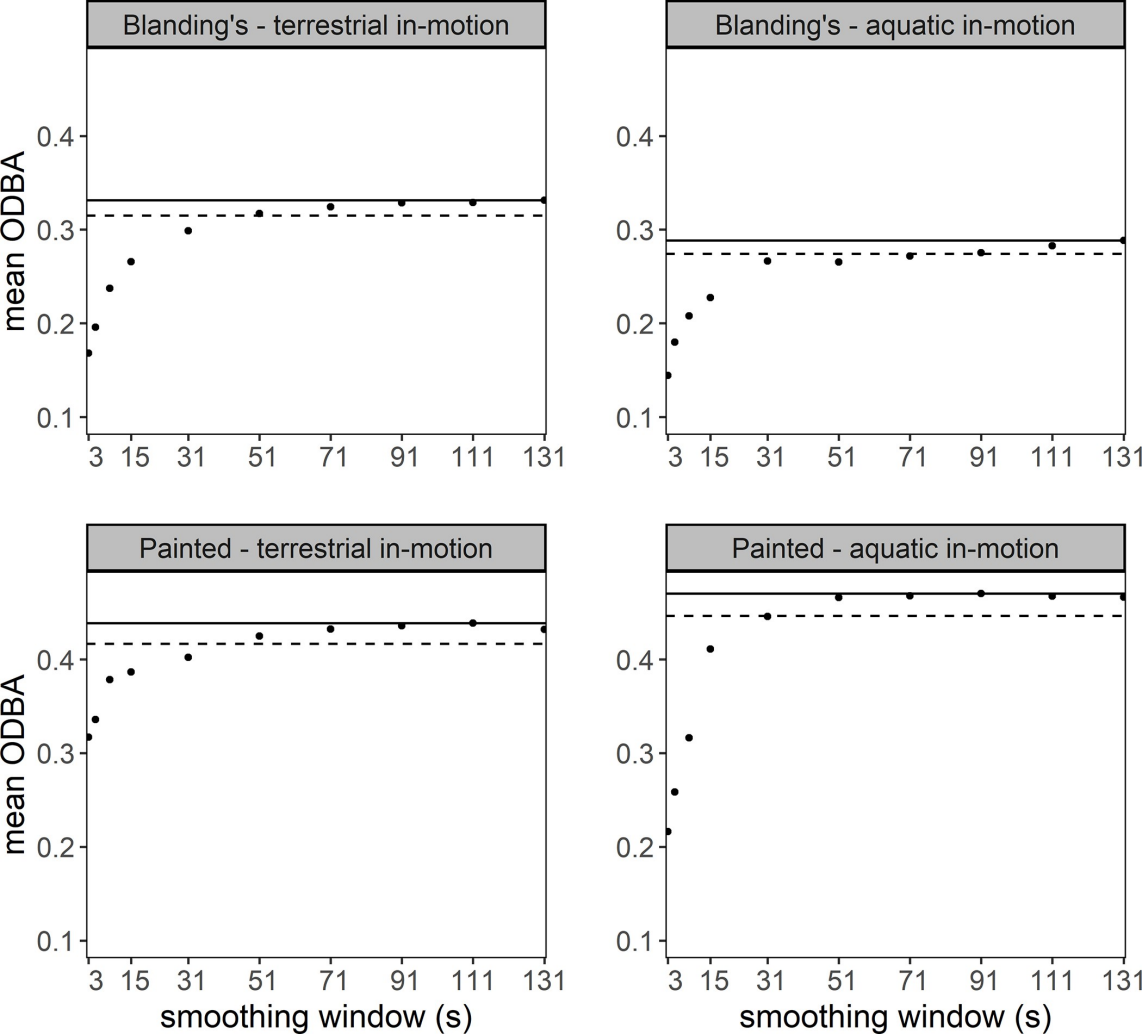
Mean overall dynamic body acceleration (ODBA) as a function of the duration of the smoothing window for Blanding’s and Painted turtle terrestrial and aquatic motion, using accelerometer data sampled at 1 Hz. Maximum ODBA value (solid line) and 95% of the maximum ODBA value (dashed) are indicated.

For Blanding’s turtles, the threshold at which ODBA stabilised for terrestrial and aquatic in-motion was 91 s, whereas for Painted turtles, ODBA stabilised at 91 s and 51 s for terrestrial and aquatic in-motion, respectively (Fig 2). After selecting the appropriate ODBA value (within 95% of the maximum value which was comparable to the next longest running mean duration), we found that for Blanding’s turtles 91 s was the best smoothing duration for both terrestrial and aquatic in-motion. For Painted turtles, the best smoothing windows were 71 s and 31 s for terrestrial and aquatic in-motion, respectively (Fig 2), of which we selected 71 s to smooth acceleration data in Painted turtles.

### Acceleration metrics and threshold values

**Fig 3:**
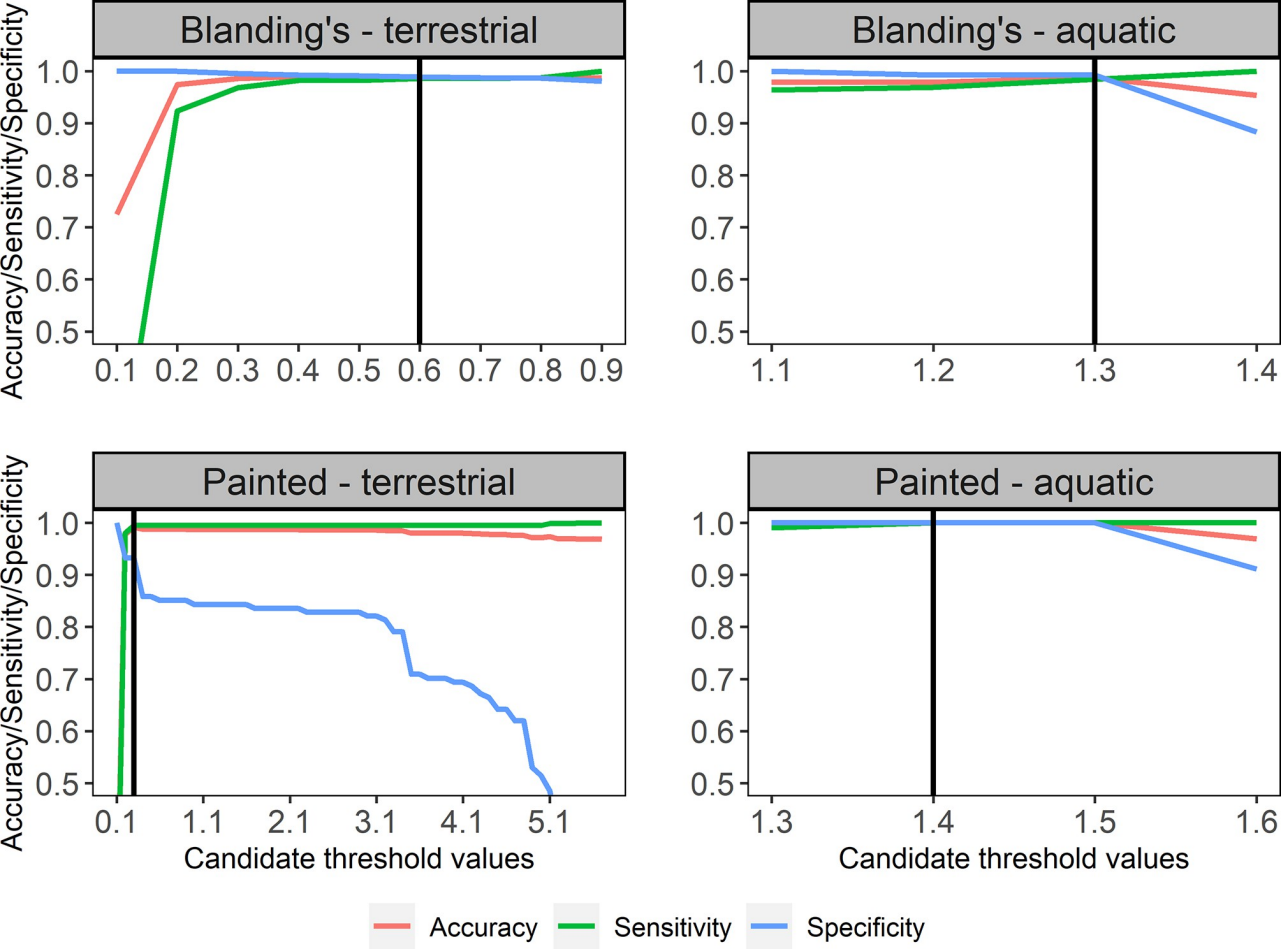
Qualitative selection of the most suitable threshold value (vertical line) relative to accuracy, sensitivity and specificity for Blanding’s turtles and Painted turtles, using accelerometer data sampled at 1 Hz.

**Original dataset.** The six acceleration metrics were all highly correlated (mean Pearson’s correlation coefficient r = 0.83, range = 0.63–1.00, S2 Table). Histogram separation of terrestrial and aquatic states indicated that generally ΔODBA and ΔVeDBA most clearly separated states in both species. Notably, ΔODBA was the DBA metric that separated aquatic states with the least overlap in both species (S1 Table, S4 Fig), which justified selection of this metric over all others. Within overlapping regions of the histograms, we tested performance of ΔODBA in assigning known activity and found that 0.6 was the best threshold separating terrestrial in-motion from motionless in Blanding’s turtles (accuracy, sensitivity, specificity: all > 98%, see Fig 3). In Blanding’s turtles, a 1.3 threshold separated aquatic in-motion from motionless (accuracy, sensitivity, specificity: all > 98%). For Painted turtles, a 0.3 threshold separated terrestrial activity (accuracy, sensitivity, specificity: > 93%, see Fig 3). We found that threshold values of 1.4 and 1.5 were comparable in separating aquatic activity for Painted turtles (accuracy, sensitivity, specificity: all 100%). We chose the more conservative threshold (1.4) because of its higher overall accuracy, sensitivity, and specificity (Fig 3).

#### Cross-species comparison

Using a smoothing window of 71 s and 91 s for Blanding’s and Painted turtles, respectively, and assessing histogram separation, ΔODBA was chosen to separate terrestrial in-motion from motionless and aquatic in-motion from motionless. In Blanding’s turtles, ΔODBA and ΔVeDBA best separated terrestrial activity, while aquatic activity was separated using ΔODBA. In Painted turtles, ΔODBA, ΔVeDBA and SDVeDBA all separated terrestrial activity comparably, with ΔODBA separating aquatic activity with the least overlap (S1 Table). Threshold values with the highest accuracy were identical to those calculated with the original smoothing windows: in Blanding’s turtles, threshold 0.6 separated terrestrial activity (all metrics > 98%) and 1.3 separated aquatic activity (all metrics > 98%, see S5 Fig). In Painted turtles, threshold 0.3 was selected to separate terrestrial activity (all metrics > 93%) and threshold 1.4 best separated aquatic activity, albeit comparably to the 1.5 threshold (all metrics > 99%, see S5 Fig).

### Performance of threshold values

#### Original dataset

Using our selected threshold values, terrestrial and aquatic activity were separated in Blanding’s turtles with 99% and 84% accuracy, respectively, and in Painted turtles with 97% and 92% accuracy (Table 1). The main sources of error were misclassifying aquatic-motionless as aquatic in-motion in Blanding’s turtles and Painted turtles (22/83 and 11/100 events, respectively), and terrestrial-motionless as terrestrial in-motion in Painted turtles (42/1136 events). Accuracy was slightly reduced with the inclusion of water sensor data (Blanding’s turtles: 92%; Painted turtles: 77%). Errors in assigning Blanding’s turtle state mainly arose from misclassifying aquatic-motionless as either terrestrial-motionless (18/83 events), aquatic in-motion (22/83 events) or terrestrial in-motion (10/83 events). Aquatic in-motion was falsely classified as terrestrial in-motion in a few instances (13/54 events) (Table 2). Errors in assigning Painted turtle state mainly arose from misclassifying terrestrial-motionless as aquatic-motionless (233/1136 events) or terrestrial in-motion (42/1136 events), as well as classifying terrestrial in-motion as aquatic in-motion (19/61 events) and aquatic-motionless as aquatic in-motion (11/100 events) (Table 2).

**Table 1:** Overall classification performance for the testing data used to classify Blanding’s turtle and Painted turtle activity based on only accelerometry data, sampled at 1 Hz.

Species	Separation of in-motion vs. motionless	Threshold	Accuracy (%) (95% CI)	Sensitivity (%)	Specificity (%)
**Blanding’s**	Terrestrial	0.6	99.3 (98.4, 99.7)	99.6	99.2
	Aquatic	1.3	83.9 (76.7, 89.7)	73.5	100
**Painted**	Terrestrial	0.3	96.5 (95.3, 97.5)	96.3	100
	Aquatic	1.4	91.8 (85.8, 95.8)	89.0	100

**Table 2:** Overall classification performance for the testing data used to classify Blanding’s turtle and Painted turtle activity based on accelerometry and water sensor data, sampled at 1 Hz.

**Blanding`s**		**Observed**				
	**Predicted**		**Motionless (aquatic)**	**Motionless (terrestrial)**	**In-motion (aquatic)**	**In-motion (terrestrial)**
		Motionless (aquatic)	33	0	0	0
		Motionless (terrestrial)	18	242	0	4
		In-motion (aquatic)	22	0	41	0
		In-motion (terrestrial)	10	1	13	464
		Sensitivity (%)	39.8	99.6	75.9	99.2
		Specificity (%)	100	96.4	97.2	93.7
		Overall accuracy (%): 92.0 (95% CI: 89.9, 93.7%)				
**Painted**			**Motionless (aquatic)**	**Motionless (terrestrial)**	**In-motion (aquatic)**	**In-motion (terrestrial)**
	**Predicted**	Motionless (aquatic)	89	233	0	0
		Motionless (terrestrial)	0	861	0	0
		In-motion (aquatic)	11	0	34	19
		In-motion (terrestrial)	0	42	0	42
		Sensitivity (%)	89.0	75.8	100	68.9
		Specificity (%)	81.1	100	97.7	96.7
		Overall accuracy (%): 77.1 (95% CI: 74.7, 79.3%)				

#### Cross-species comparison

Cross-species comparison of activity classification revealed high transferability between the two species. Painted turtle thresholds classified Blanding’s turtle terrestrial and aquatic activity with 99% and 85% accuracy, respectively. Blanding’s turtle thresholds separated Painted turtle terrestrial and aquatic activity with 98% and 91% accuracy, respectively (Table 3). When including water sensor data in the model, overall accuracy of Blanding’s turtle classification using Painted turtle thresholds was 93%, with sources of error being due to misclassification of aquatic-motionless as either terrestrial-motionless (6/83 events) or aquatic in-motion (20/83). Painted turtle classification model accuracy when using Blanding’s turtle thresholds was 78%, with main misclassifications being due to assigning aquatic-motionless to terrestrial-motionless (233/1136), terrestrial in-motion to terrestrial-motionless (25/1136) and aquatic in-motion to aquatic-motionless (12/100) (S3 Table).

**Table 3:** Classification performance for the testing data used to classify Blanding’s turtle and Painted turtle activity based on accelerometry data, using the other species’ threshold values.

Species	Separation of In-motion vs. motionless	Threshold	Accuracy (%) (95% CI)	Sensitivity (%)	Specificity (%)
**Blanding’s**	Terrestrial	0.3	99.4 (98.6, 99.9)	99.6	99.4
	Aquatic	1.4	85.2 (78.4, 91.0)	75.9	100
**Painted**	Terrestrial	0.6	97.9 (96.9, 98.6)	97.8	100
	Aquatic	1.3	91.0 (84.8, 95.3)	89.0	100

#### Effect of sampling frequency

Smoothing windows using rarefied datasets were generally longer compared to the 1 Hz dataset in both species, except for 0.5 Hz Painted turtle data, which was slightly shorter (see S4 Table). Threshold values separating states using 0.5 and 0.25 Hz datasets were only marginally different from the original dataset, but were 17–75% higher using 0.125 and 0.0625 Hz datasets (see S4 Table). Similarly to the original dataset, we found that ΔODBA effectively separated terrestrial and aquatic activity in both species using all rarefied datasets. Lastly, we found that accuracy measurements of activity classification did not decrease with lower sampling frequencies. Accuracy of the two-branch decision tree (including both accelerometer and water sensor data) ranged from 79.4 to 91.9% (mean = 87.5%), when using sampling frequencies of 0.5, 0.25, 0.125 and 0.0625 Hz (see S4 Table).

#### Activity-budgets

**Fig 4:**
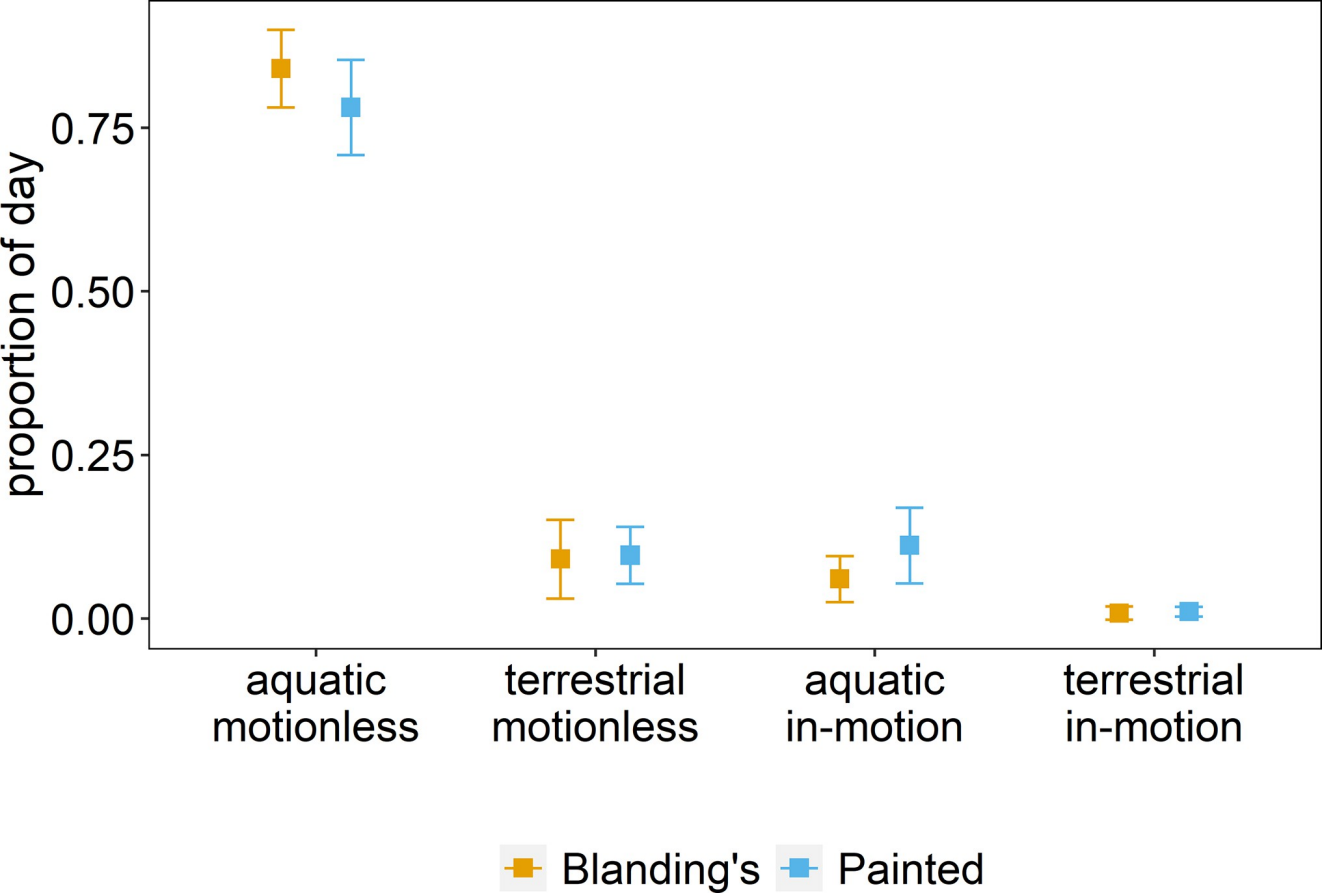
Daily activity-budget for Blanding’s (n = 16) and Painted turtles (n = 23) in the South March Highlands, Ottawa. Shown are mean proportion (± SD) of time spent doing each of the four main states during a 24-hour period.

Time-activity budgets during 2018–2020 (days monitored: Blanding’s turtles: mean = 84.3, range: 8–164 per individual; Painted turtles: mean = 74.8, range: 20 – 159 per individual) revealed that species exhibited mostly similar proportions of activity in both aquatic and terrestrial environments, with only modest differences in time allocation (Fig 4): Both species spent most of their time motionless, with Blanding’s turtles spending 84.0% (± SD 5.9%) and Painted turtles 78.1% (± 7.3%) of their day motionless under water (Dirichlet z-value = 0.647, p = 0.517); whereas Blanding’s turtle spent 9.1% (± 6.0%) and Painted turtles 9.7% (± 4.4%) motionless on land (z-value = 1.528, p = 0.127). In contrast, Blanding’s turtles spent 6.0% (± 3.5%) and Painted turtles 11.2% (± 5.8%) of the day in-motion under water (z-value = 2.511, p = 0.012). In-motion on land occurred rarely, with Blanding’s turtles spending 0.8% (± 1.0) and Painted turtles 1.0% (± 0.7) of the time engaging in terrestrial activity (z-value = 1.294, p = 0.196) (Fig 4).

## Discussion

Using a combination of accelerometers and water sensors, we classified activity of two free-ranging freshwater turtle species with high accuracy and achieved higher predictive accuracy when differentiating activity on land or in water separately using only accelerometer data (> 83%) than when also considering water sensor data (accuracy > 75%). Our model accuracy was comparable to studies classifying behaviours in other species exhibiting relatively simple behavioural repertoires (e.g. [50, 51]), and in general threshold values separating terrestrial and aquatic states were higher in the former environment. Interestingly, minor differences in classification threshold values between the two species did not impact the transferability of models between species, allowing us to conclude that accelerometry holds promise for broadly classifying activity of free-ranging freshwater turtles. Unsurprisingly, our simple behavioural classification was similarly successful when sampling frequency was reduced by 16-fold. Our results demonstrate high accuracy in classifying simple activity states as well as cross-species transferability of classification models among closely-related species in similar environments. Our research adds to other accelerometer-based behavioural identification studies, such as those by Marchand et al. [52] and Lagarde et al. [53], which describe fine-scaled behaviour in European pond turtle (*Emys orbicularis*) and Greek tortoises (*Testudo graeco*), respectively. Our research expands on these studies by characterising activity in two species of free-ranging turtles in their wild, native environment, which may be more representative of and transferable to real-life behavioural data collected by many researchers.

A primary objective in calibrating accelerometry data is to determine the appropriate smoothing window to classify activity states and behaviours. For Blanding’s turtles, the longer smoothing window can be explained by their larger body size and thus greater stroke length compared to Painted turtles [54]. This is consistent with the results of Shepard et al. [35] that show a positive relationship between stroke length and the running mean at which ODBA stabilised. For Painted turtles, we selected the longer plausible smoothing window to avoid underestimating the dynamic portion of acceleration (see [35]). Generally, our smoothing windows were longer than others used in mammal or bird behavioural calibrations, which often ranged from 2–4 s (e.g. [24, 31, 35]); we infer this difference as being the result of slow movement and therefore long stroke length of our study animals relative to their body size. Regardless, despite small differences in optimal smoothing windows between our study species, cross-species validation suggests that these differences do not necessarily affect threshold values separating states or accuracy of activity predictions. While broad activity states in our study system may be separated based on raw acceleration signatures in each of the 3 body axes (see S1 Fig), we selected ODBA as the metric to define behaviours. ODBA is the most prevalent metric in the accelerometer literature, and its correlation with VeDBA has been demonstrated previously (e.g. [40, 55]). Accordingly, our choice of ODBA was appropriate and it seems that this metric will be well-suited for a wide range of species that are tracked via accelerometers [19, 22].

While direct comparisons of accelerometer-derived behavioural signatures between species are rare in the literature, our findings are consistent with other studies showing minor and largely negligible influence of body size and device attachment on accelerometer readings [31, 56]. For example, the observation that terrestrial activity in Blanding’s turtles was separated by greater thresholds than in Painted turtles is likely due to the larger carapace of the former species. When accelerometers are attached to the carapace margin of the larger species, even small body movements might translate to higher acceleration due to a greater distance to the center of mass. Further, misclassification of in-water vs. out-of-water between species may be explained by relatively flat carapaces of Painted turtles requiring the water sensor to be mounted lower on the shell than for Blanding’s turtles, and resulting in Painted turtles being recorded as using aquatic habitat at shallower depths. Even though in our study system differences in threshold values between species due to turtle body shapes and sizes did not impact transferability of classification models between species, our findings highlight the need for careful placement of sensors, especially where accurate separation between aquatic and terrestrial activity is a high priority. Arguably, attaching the device on the top of the carapace would produce more accurate sensor readings [52], and could ultimately improve classification performance. However, this option would likely increase the risk of device loss (A. Auge, unpubl.). Generally, larger ΔODBA thresholds separating motion from motionless states in aquatic compared to terrestrial environments is comparable to other studies showing that waves and water currents can lead to variation in measured acceleration due to passive motion [57]. This effect is stronger in lighter animals, resulting in higher thresholds in aquatic (but not terrestrial) habitat for Painted turtles. Other studies emphasize the need to consider the contribution of water currents or wind to accelerometer readings [58, 59], and our results confirmed that comparable activity can vary in accelerometer signatures between environments, and thus require separate examination and validation across habitats. Nonetheless, despite decreased accuracy of the more complex classification model, including more than one bio-sensor is usually preferred as it allows the description of much broader ecological contexts of behaviours [22]. We note that our manual approach in developing classification models allowed us to investigate these often subtle differences in acceleration signatures between activity states, which may be less transparent in other approaches, such as unsupervised machine learning [26, 29].

Cross-species fitting accelerometer-based activity classification models is an important step to assess the generality of such models. While not commonly tested, some studies have also found high transferability of behavioural classification models between closely related species (e.g. [60]), while others have not (e.g. [61]). For example, a case study assessing behavioural classification performance in wolves (*Canis lupus*) and domestic dogs (*Canis familiaris*) found lower cross-species model accuracy (≤ 51%) than what we observed for freshwater turtles [32]. This difference could be related to the more restricted suite of behaviours under consideration and larger distinction in accelerometer readings between states in our study. Regardless, our findings are important because they show that deriving a single classification tree across similar species holds promise for improving model development, by streamlining the classification process and potentially applying one model across a variety of species–instead of conducting costly validation on multiple species. However, it should be noted that classification models are likely only interchangeable when accelerometer devices are identical and device position is consistent. Indeed, our preliminary trials using different accelerometers from two manufacturers, and even using different device models from the same manufacturer, yielded > 10% variation in activity classification (A. Auge, pers. obs., see also [34]. Therefore, researchers should only consider cross-species application of classification models for comparable devices and species with similar behavioural traits, and only after robust testing and validation.

It is not especially surprising that lower sampling frequencies up to 0.0625 Hz yields equally reliable activity information for slow-moving animals like freshwater turtles [35], thereby supporting findings from other studies showing classification success at similarly low sampling frequencies (e.g. [27]). While few studies have investigated the impacts of a range of sampling frequencies on classification performance [33, 47], none so far, to our knowledge, have assessed the effect of very low (< 1Hz) frequencies. Assessing the impact of a range of sampling frequencies on the performance of activity classification is an important step in studying behaviour of wild animals using accelerometers, as it allows refinement of accelerometer settings before deployment. High classification performance at low frequencies could allow longer battery life, increased memory capacity, and, thus, longer field deployment duration [33, 62]. In addition, low-frequency accelerometer data require lower computational power for processing and analysis [56]. It is important to note, however, that species exhibiting behaviours with complex and fast kinematics, may require high-frequency accelerometry for reliable inference and representation [22, 54].

Our study provides a robust framework for rigorously testing the suitability of accelerometers for behavioural research in ecology. We conclude that accelerometers and other bio-logging tools hold much promise for characterising activity levels in free-ranging animals as well as developing behavioural profiles of cryptic and elusive species [22, 52, 63]. Accordingly, with proper validation measures such as those outlined herein, we expect accelerometry to become increasingly valuable as a tool for tracking animal behaviour across a variety of research and conservation or management contexts [64].

## Supporting information

S1 Table Correlation matrix showing Pearson’s correlation coefficients between different accelerometer metrics. All correlations were significant (p < 0.001).
(PDF)

S2 Table Percent overlap of the best and second-best accelerometer metrics following histogram separation of terrestrial and aquatic in-motion and motionless. (PDF)

S3 Table State assignment accuracy for the testing data used to classify Blanding’s turtle and Painted turtle accelerometry and water sensor data, using the other species threshold values. (PDF)

S4 Table Effect of sampling frequency on state classification accuracy. Best-fit smoothing window, threshold values, accuracy, sensitivity and specificity of Blanding’s turtle and Painted turtle activity assignment based on accelerometry data, sampled at 0.5, 0.25, 0.125 and 0.0625 Hz.
(PDF)

S1 Fig Example of raw acceleration in each of the three body axes (surge, sway, heave) for each of the four activity states, and the orientation of the accelerometer on the turtle carapace margin (note that sizes are not to scale). (PDF)

S2 Fig Accelerometer (left) and VHF transmitter (right) bolted onto the rear carapace margin of a Painted turtle.
(PDF)

S3 Fig Boxplot of length of recorded states (top panel), and number of occasions each state was observed (bottom panel) across Blanding’s (blue) and Painted turtles (orange).
(PDF)

S4 Fig Sample histogram separating activity modes in Blanding’s turtles: Terrestrial in-motion from motionless and aquatic in-motion from motionless. The red vertical line indicates the threshold value determined after testing the accuracy of ΔODBA values within the overlapping regions. These histograms are based on data sampled at 1 Hz.
(PDF)

S5 Fig Optimizing the threshold value (vertical lines) relative to data accuracy, sensitivity and specificity, for Blanding’s turtles and Painted turtles, using acceleration data calculated with smoothing windows of the other species. (PDF)
