# Supplementary material for: Developing a classification system to assign activity states to two species of freshwater turtles
Source: PLoS One. 2022 Nov 30;17(11):e0277491. doi: 10.1371/journal.pone.0277491 (PMC9710770; doi:10.1371/journal.pone.0277491)
Supplement: S1 Table — All correlations were significant (p < 0.001). (PDF) [file pone.0277491.s001.pdf]

S1 Table

	ODBA	VeDBA	ΔODBA	ΔVeDBA	TODBA	TVeDBA	SDODBA	SDVeDBA
ODBA	1.00							
VeDBA	0.99	1.00						
ΔODBA	0.65	0.65	1.00					
ΔVeDBA	0.65	0.65	1.00	1.00				
TODBA	0.78	0.79	0.85	0.85	1.00			
TVeDBA	0.78	0.79	0.84	0.84	1.00	1.00		
SDODBA	0.64	0.63	0.81	0.81	0.70	0.69	1.00	
SDVeDBA	0.64	0.64	0.82	0.82	0.71	0.71	0.99	1.00
