# Supplementary material for: Developing a classification system to assign activity states to two species of freshwater turtles
Source: PLoS One. 2022 Nov 30;17(11):e0277491. doi: 10.1371/journal.pone.0277491 (PMC9710770; doi:10.1371/journal.pone.0277491)
Supplement: S2 Table — (PDF) [file pone.0277491.s002.pdf]

S2 Table

Dataset	Species	Separated activities	Best summary statistic	% overlap	% improvement to second best statistic	Second best summary statistic
1 Hz sampling frequency	Blanding's	Terrestrial-in-motion vs. motionless	ΔODBA, ΔVeDBA	1.4	61.1	SDODBA
		Aquatic-in-motion vs. motionless	ΔODBA	6.7	5.6	ΔVeDBA
	Painted	Terrestrial-in-motion vs. motionless	ΔODBA, ΔVeDBA	1.1	9	TODBA
		Aquatic-in-motion vs. motionless	ΔODBA	0.5	16.7	ΔVeDBA
Cross-species comparison	Blanding's	Terrestrial-in-motion vs. motionless	ΔODBA, ΔVeDBA	1.6	38.5	TODBA
		Aquatic-in-motion vs. motionless	ΔODBA	6.2	3.1	ΔVeDBA
	Painted	Terrestrial-in-motion vs. motionless	ΔODBA, ΔVeDBA, SDVeDBA	1.1	8.3	TODBA
		Aquatic-in-motion vs. motionless	ΔODBA	0.6	14.3	ΔVeDBA
