# Supplementary material for: Developing a classification system to assign activity states to two species of freshwater turtles
Source: PLoS One. 2022 Nov 30;17(11):e0277491. doi: 10.1371/journal.pone.0277491 (PMC9710770; doi:10.1371/journal.pone.0277491)
Supplement: S3 Table — (PDF) [file pone.0277491.s003.pdf]

S3 Table

			Observed			
			Motionless (aquatic)	Motionless (terrestrial)	In-motion (aquatic)	In-motion (terrestrial)
Blanding`s	Predicted	Motionless (aquatic)	35	0	0	0
		Motionless (terrestrial)	6	242	0	3
		In-motion (aquatic)	20	0	41	0
		In-motion (terrestrial)	22	1	13	465
		Sensitivity (%)	42.2	99.6	75.9	99.4
		Specificity (%)	100	98.5	97.5	90.5
		Overall accuracy (%):	92.5 (95% CI: 90.3, 94.0%)			
			Motionless (aquatic)	Motionless (terrestrial)	In-motion (aquatic)	In-motion (terrestrial)
Painted	Predicted	Motionless (aquatic)	88	233	0	0
		Motionless (terrestrial)	0	878	0	0
		In-motion (aquatic)	12	0	34	19
		In-motion (terrestrial)	0	25	0	42
		Sensitivity (%)	88.0	77.0	100	68.9
		Specificity (%)	81.1	100	97.6	98.0
		Overall accuracy (%):	78.3 (95% CI: 76.0, 80.5%)			
