# Supplementary material for: Developing a classification system to assign activity states to two species of freshwater turtles
Source: PLoS One. 2022 Nov 30;17(11):e0277491. doi: 10.1371/journal.pone.0277491 (PMC9710770; doi:10.1371/journal.pone.0277491)
Supplement: S4 Table — Best-fit smoothing window, threshold values, accuracy, sensitivity and specificity of Blanding’s turtle and Painted turtle activity assignment based on accelerometry data, sampled at 0.5, 0.25, 0.125 and 0.0625 Hz. (PDF) [file pone.0277491.s004.pdf]

S4 Table

**SF** (Hz)	**Species**	**Separation of in-motion vs. motionless**	**SW (s)**	**Thresh-old**	**Acc. (%) (95% CI)**	**Sens. (%)**	**Spec. (%)**	Overall accuracy (%) (**95% CI)**
**0.5**	Blanding's	Terrestrial	90	0.7	99.2 (97.6, 99.8)	99.2	99.2	93.2 (90.3, 95.4)
		Aquatic		1.2	92.9 (84.1, 97.6)	88.1	100	
	Painted	Terrestrial	50	0.4	98.3 (97.0, 99.2)	98.3	100	79.4 (76.1, 82.4)
		Aquatic		1.5	98.5 (92.0, 1.0)	98.0	100	
**0.25**	Blanding's	Terrestrial	92	0.7	97.7(94.3, 99.4)	95.0	99.1	91.9 (87.4, 95.2)
		Aquatic		1.4	73.5 (55.6, 87.1)	70.0	78.6	
	Painted	Terrestrial	72	0.6	99.6 (98.1, 99.9)	100	92.6	91.4 (88.0, 94.3)
		Aquatic		2.1	85.3 (68.9, 95.1)	82.6	90.9	
**0.125**	Blanding's	Terrestrial	112	0.8	93.1 (85.6, 97.4)	96.7	91.2	85.8 (77.7, 91.9)
		Aquatic		1.5	73.7 (48.8, 90.9)	54.5	100	
	Painted	Terrestrial	112	1.7	96.6 (92.3, 98.9)	100	37.5	89.2 (83.4, 93.4)
		Aquatic		1.9	82.3 (56.7, 96.2)	83.3	80.0	
**0.0625**	Blanding's	Terrestrial	128	0.7	86.4 (72.3, 94.5)	68.8	96.4	80.4 (66.8, 90.2)
		Aquatic		5.1	42.9 (1.0, 81.6)	20.0	100	
	Painted	Terrestrial	96	0.9	97.3 (90.6, 99.7)	100	50	89.0 (80.2, 94.9)
		Aquatic		2.8	100 (63.1, 100)	100	100	

SF: sampling frequency, SW: smoothing window, Acc.: accuracy, Sens.: Sensitivity, Spec.: Specificity, CI: 95% confidence interval
