# Supplementary figures and images for: Developing a classification system to assign activity states to two species of freshwater turtles
Source: PLoS One. 2022 Nov 30;17(11):e0277491. doi: 10.1371/journal.pone.0277491 (PMC9710770; doi:10.1371/journal.pone.0277491)

S1 Figure

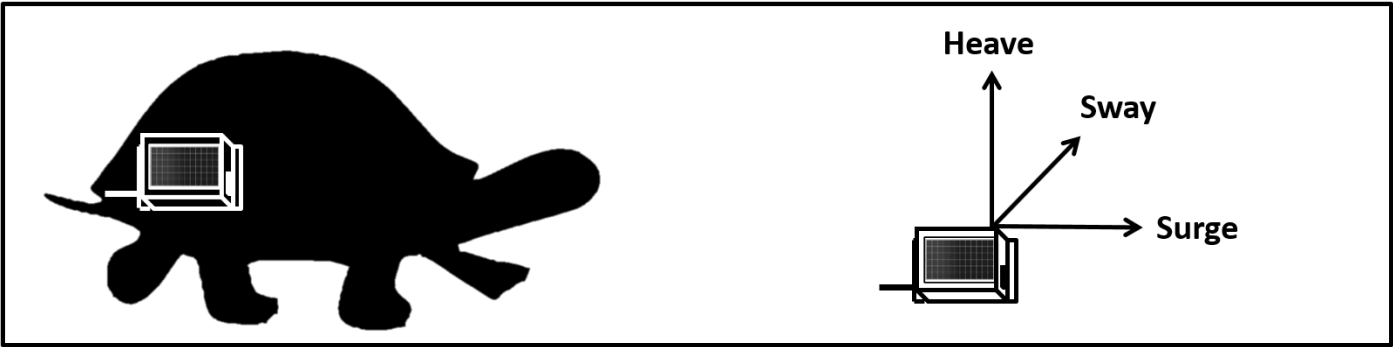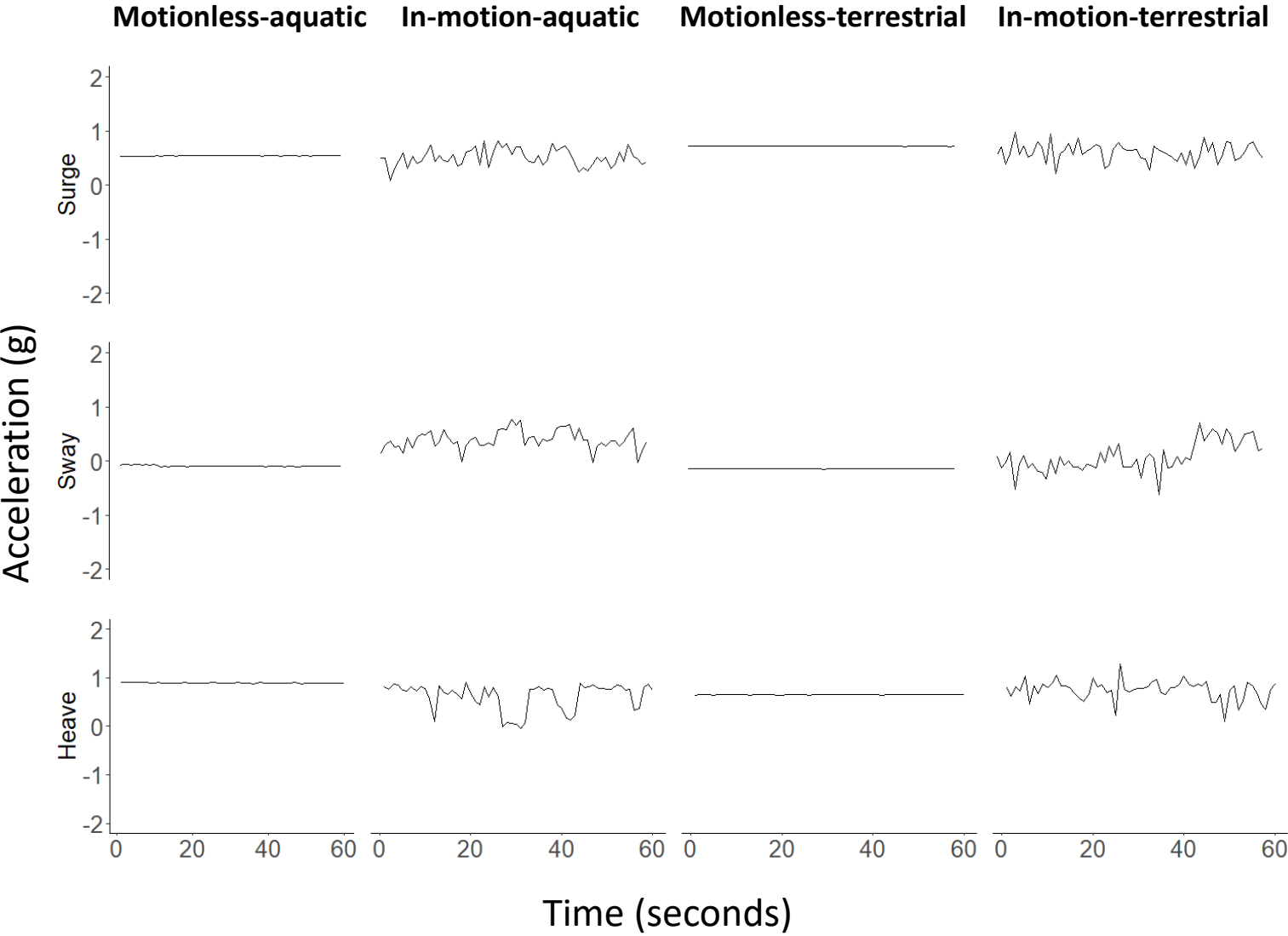

Supplement: S1 Fig — (PDF) [file pone.0277491.s005.pdf]

S2 Figure

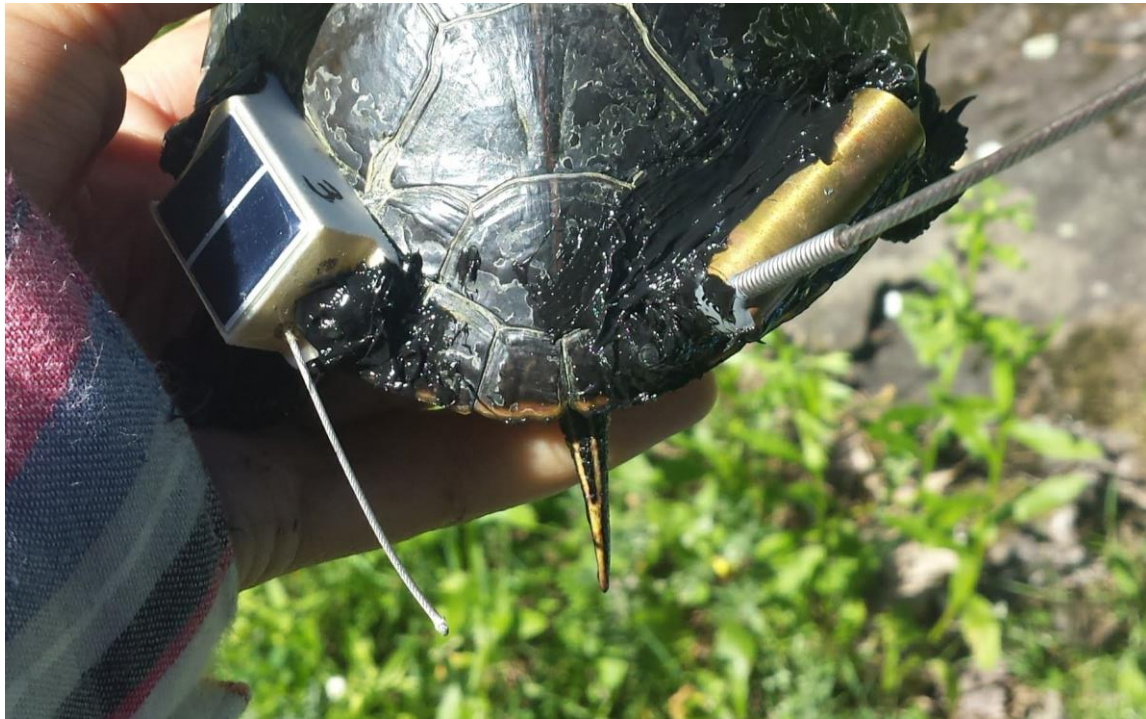

Supplement: S2 Fig — Accelerometer (left) and VHF transmitter (right) bolted onto the rear carapace margin of a Painted turtle. (PDF) [file pone.0277491.s006.pdf]

S3 Figure

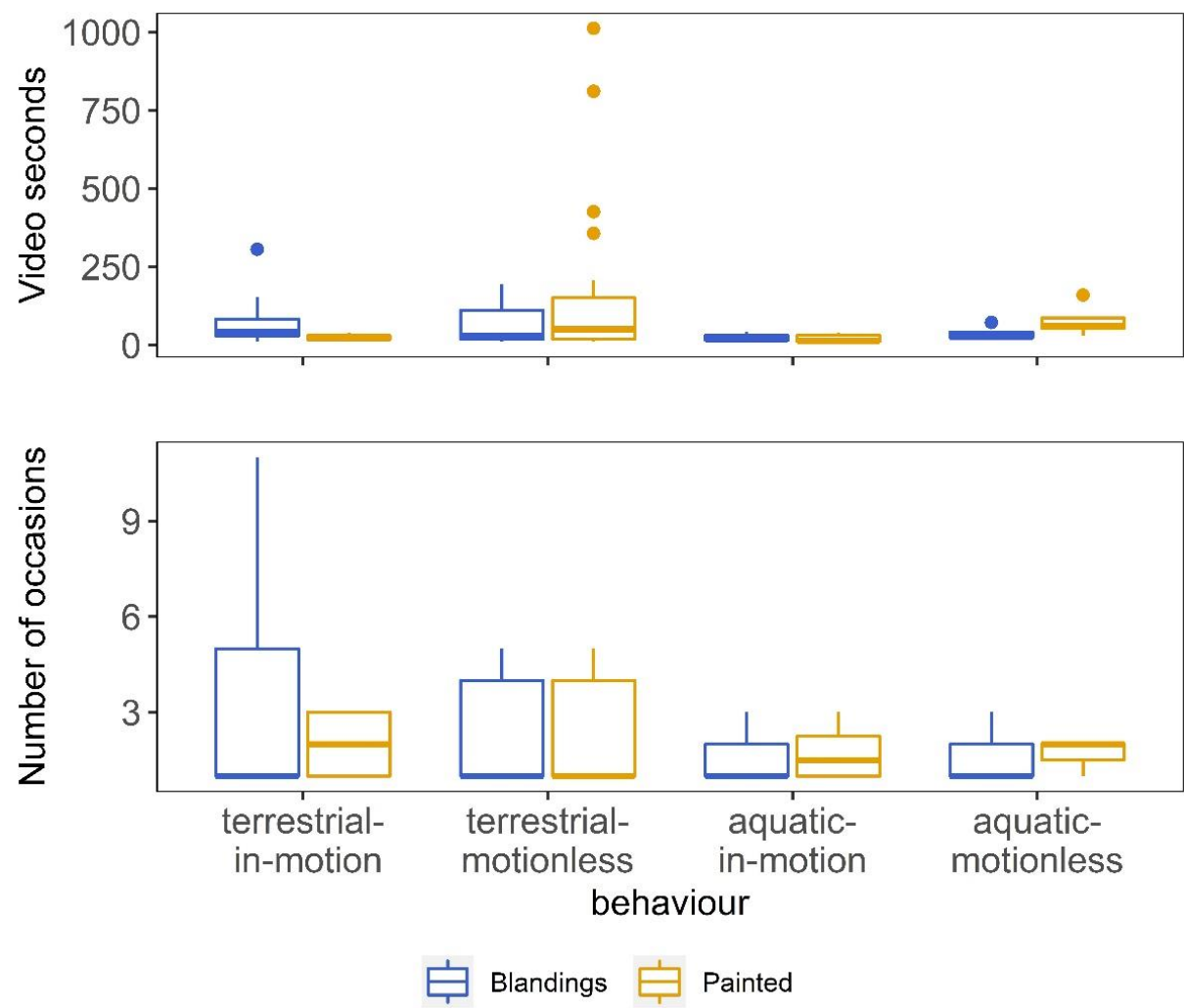

Supplement: S3 Fig — Boxplot of length of recorded states (top panel), and number of occasions each state was observed (bottom panel) across Blanding’s (blue) and Painted turtles (orange). (PDF) [file pone.0277491.s007.pdf]

S4 Figure

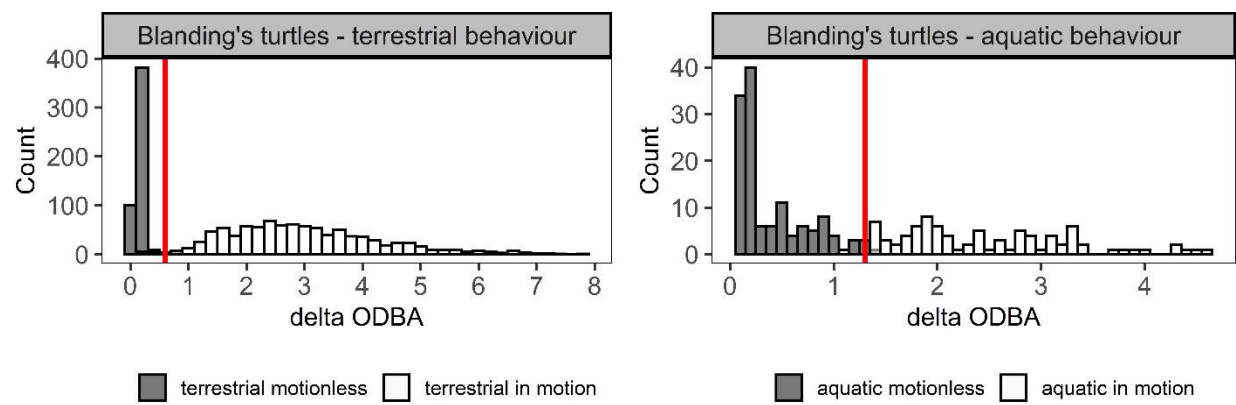

Supplement: S4 Fig — The red vertical line indicates the threshold value determined after testing the accuracy of ΔODBA values within the overlapping regions. These histograms are based on data sampled at 1 Hz. (PDF) [file pone.0277491.s008.pdf]

S5 Figure

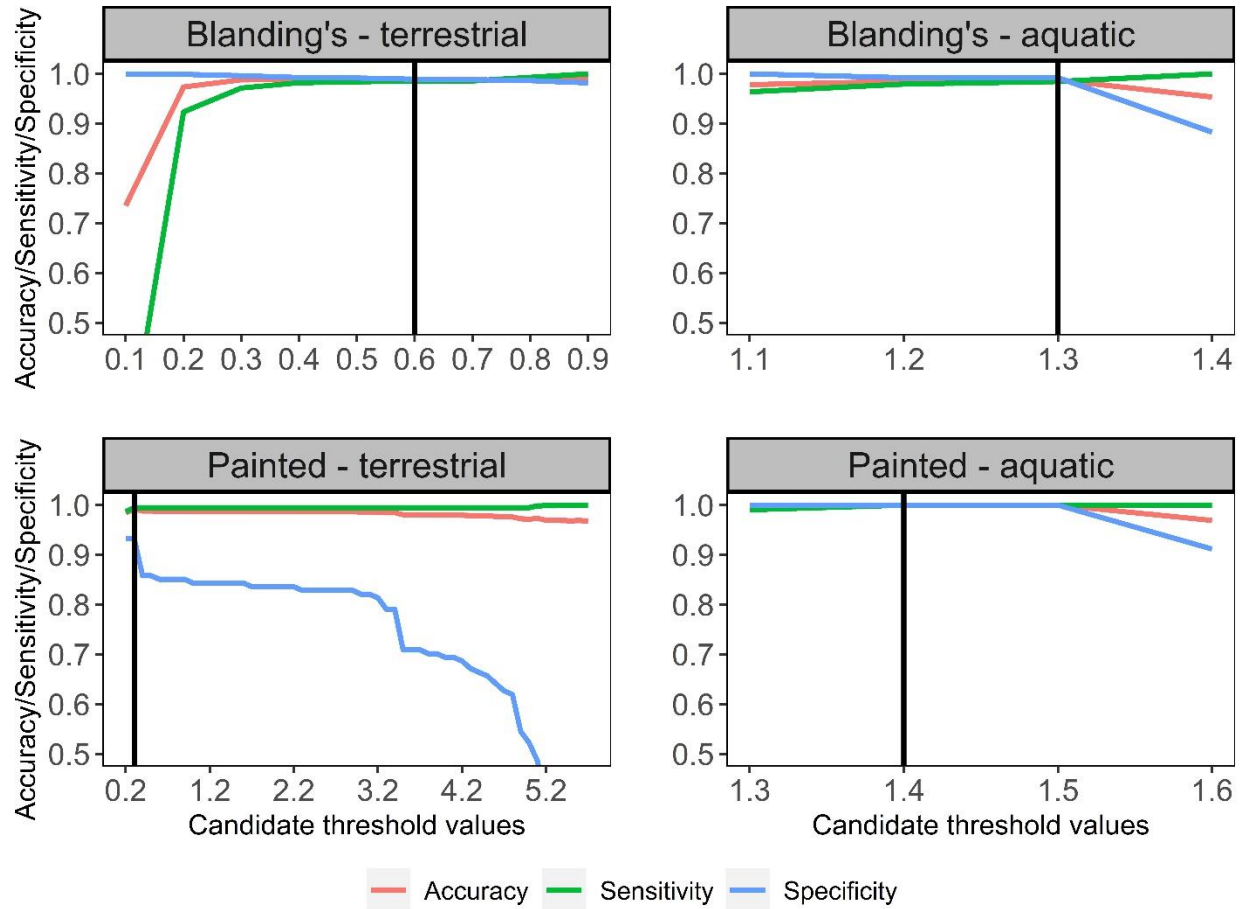

Supplement: S5 Fig — (PDF) [file pone.0277491.s009.pdf]
